# Supplementary figures and images for: Rewiring of PDZ Domain-Ligand Interaction Network Contributed to Eukaryotic Evolution
Source: PLoS Genet. 2012 Feb 9;8(2):e1002510. doi: 10.1371/journal.pgen.1002510 (PMC3276551; doi:10.1371/journal.pgen.1002510)

Quantitative model

Experimental data

MAG11\_2

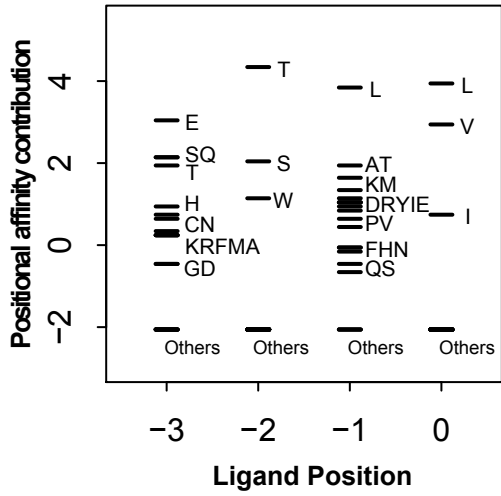

PWM similarity  
PCC = 0.85

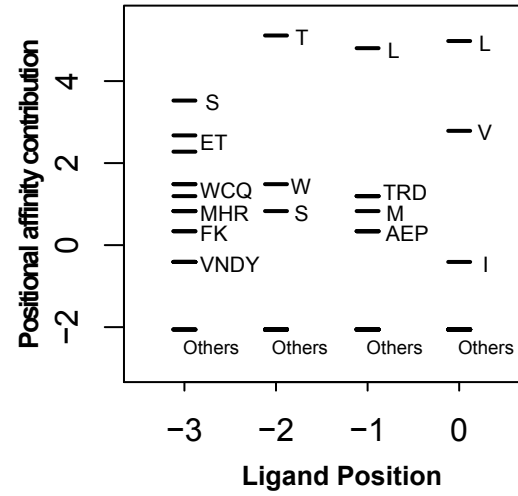

DLG1\_2

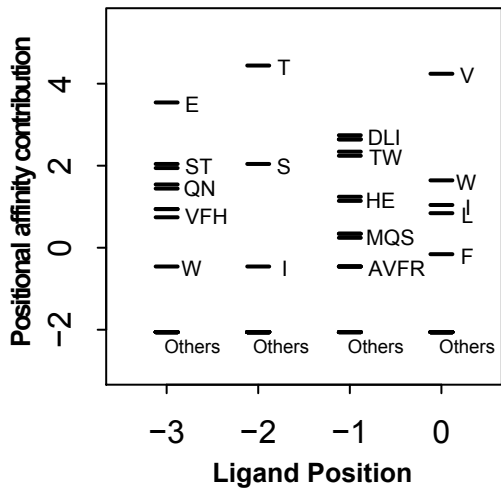

PCC = 0.84

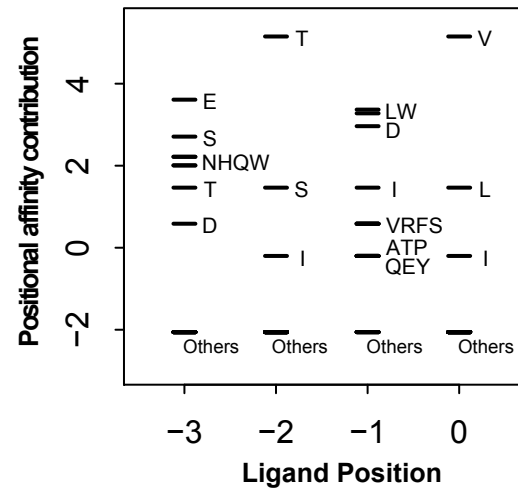

PTN13\_2

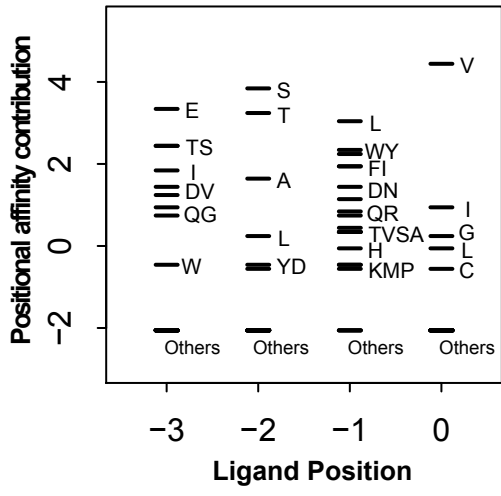

PCC = 0.78

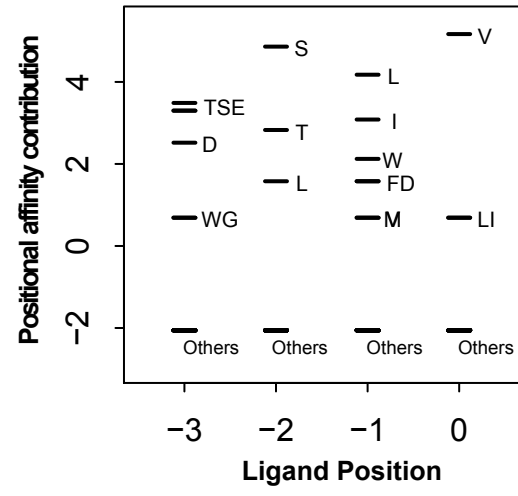

Supplement: Figure S1 — Comparisons of the quantitative model- and phage display data-derived PWMs of MAGI1_2, DLG1_2, and PTN13_2. (PDF) [file pgen.1002510.s001.pdf]

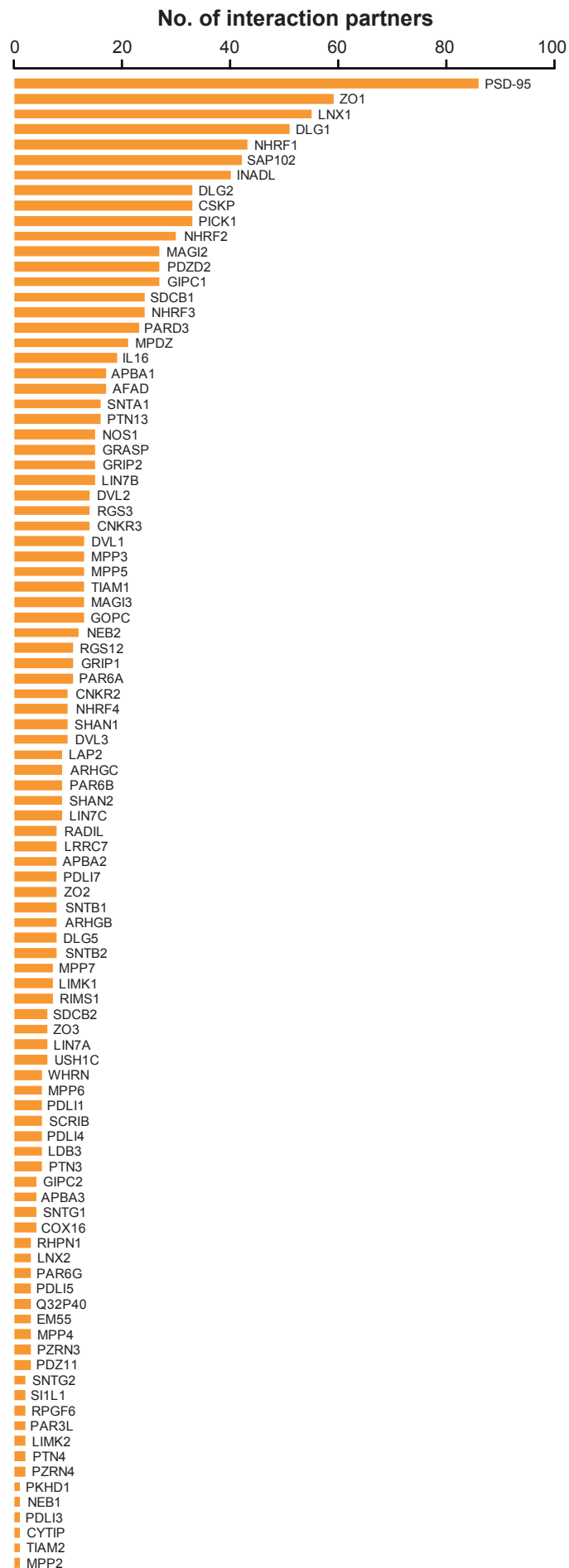

Supplement: Figure S2 — Distribution of the interaction partners of 97 human PDZ proteins. The maximum number of ligands per PDZ protein is 102. The average interaction partner of the human PDZ protein is 12. (PDF) [file pgen.1002510.s002.pdf]

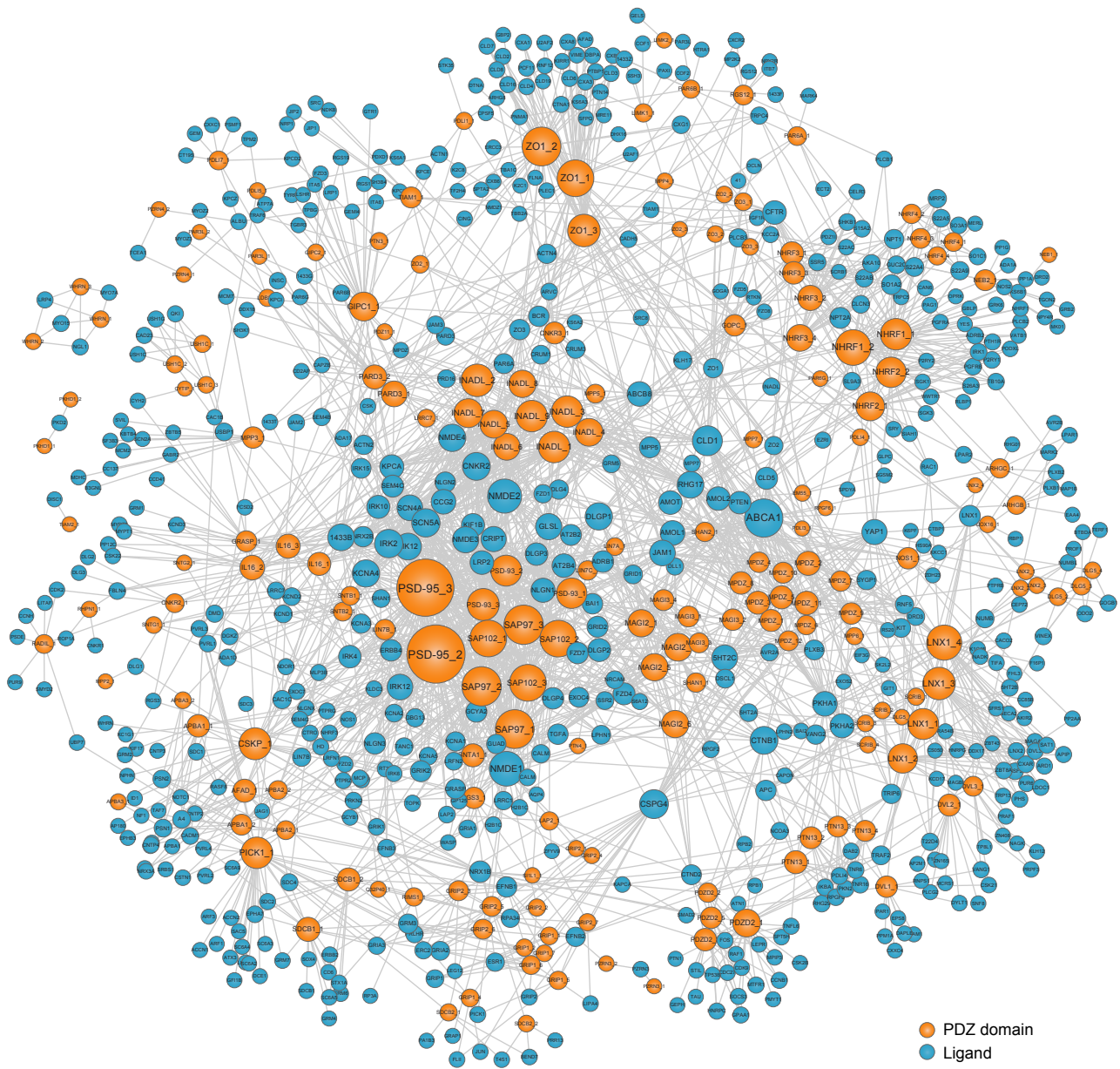

Supplement: Figure S3 — Network representation of domain-level interactions in PDZNet (best viewed by magnification in a PDF viewer). Domain numbers are presented on the right side of the PDZ protein names with a delimiter (‘_’). The network is composed of 2,643 interactions between 190 PDZ domains and 593 ligands. (PDF) [file pgen.1002510.s003.pdf]

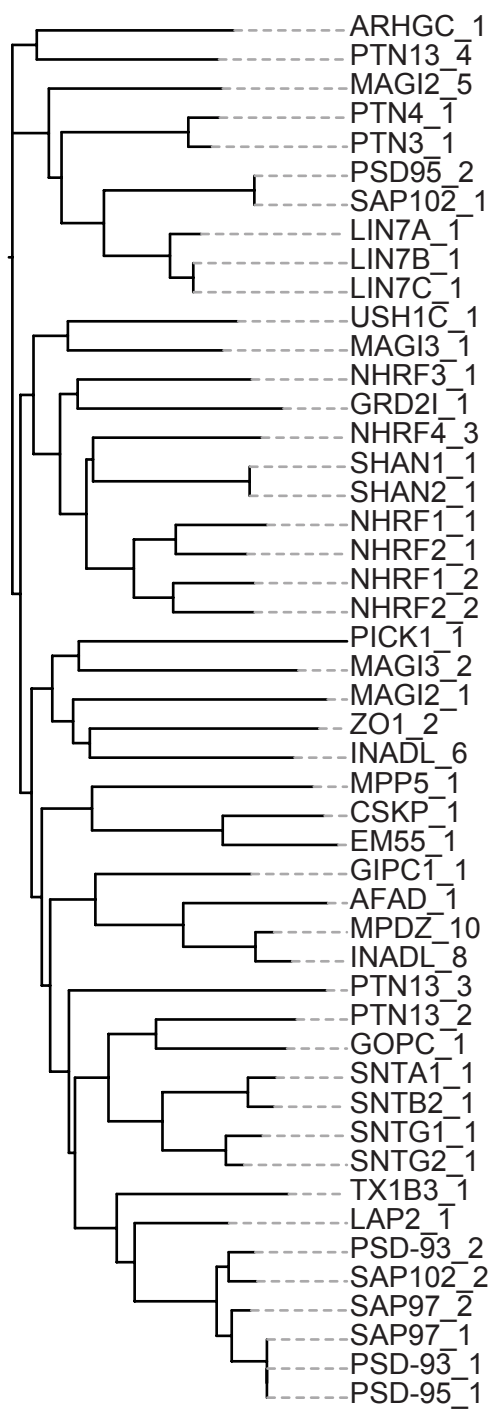

Supplement: Figure S4 — Dendrogram of PDZ domains based on the identity of pocket residues. Domain numbers are presented on the right side of the PDZ protein names with a delimiter (‘_’). (PDF) [file pgen.1002510.s004.pdf]

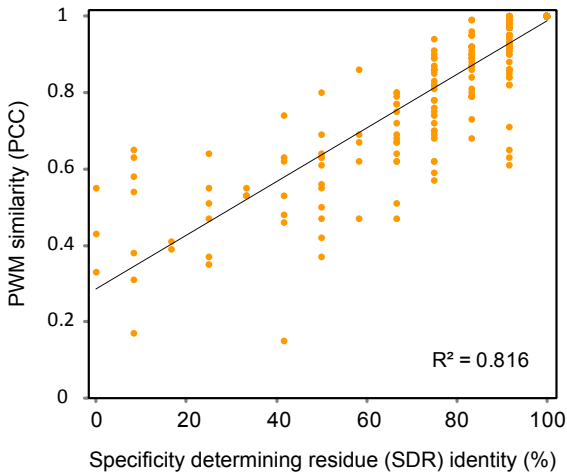

Supplement: Figure S5 — Relationship between specificity determining residue (SDR) identity and PWM similarity. Each point represents an orthologous PDZ domain pair. (PDF) [file pgen.1002510.s005.pdf]

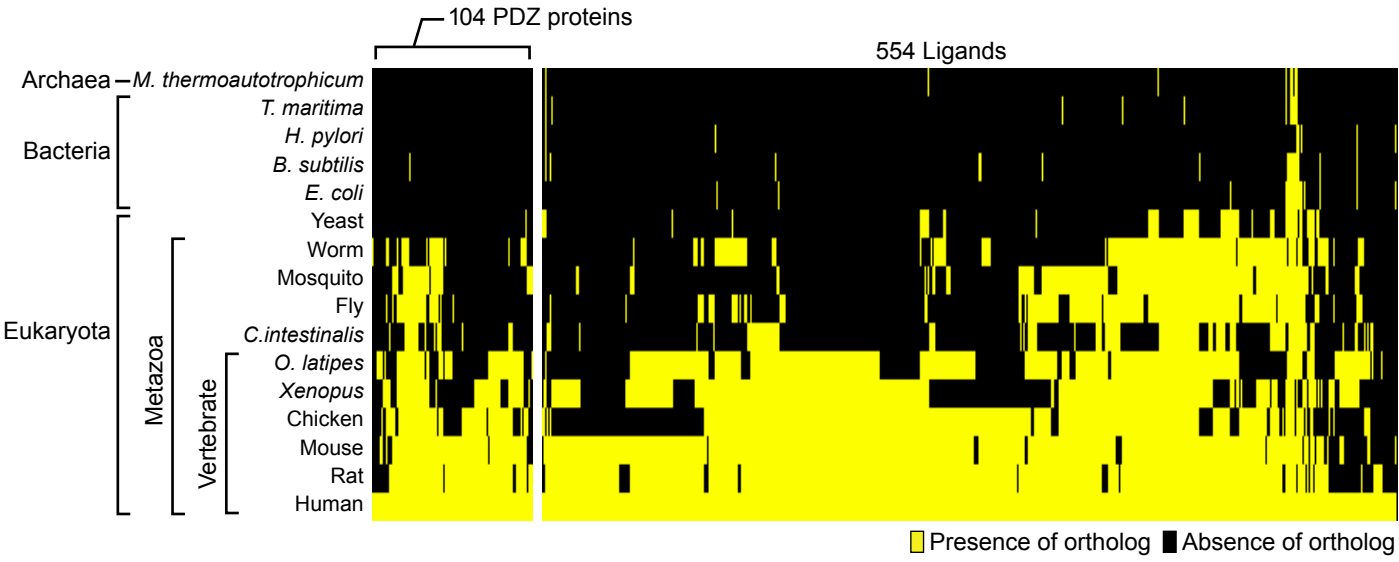

Supplement: Figure S6 — Phylogenetic profile of human PDZ proteins and ligands across 13 fully sequenced species. The presence (yellow) and absence (black) of orthologs for the 104 PDZ proteins and 554 PDZ ligands are presented. (PDF) [file pgen.1002510.s006.pdf]

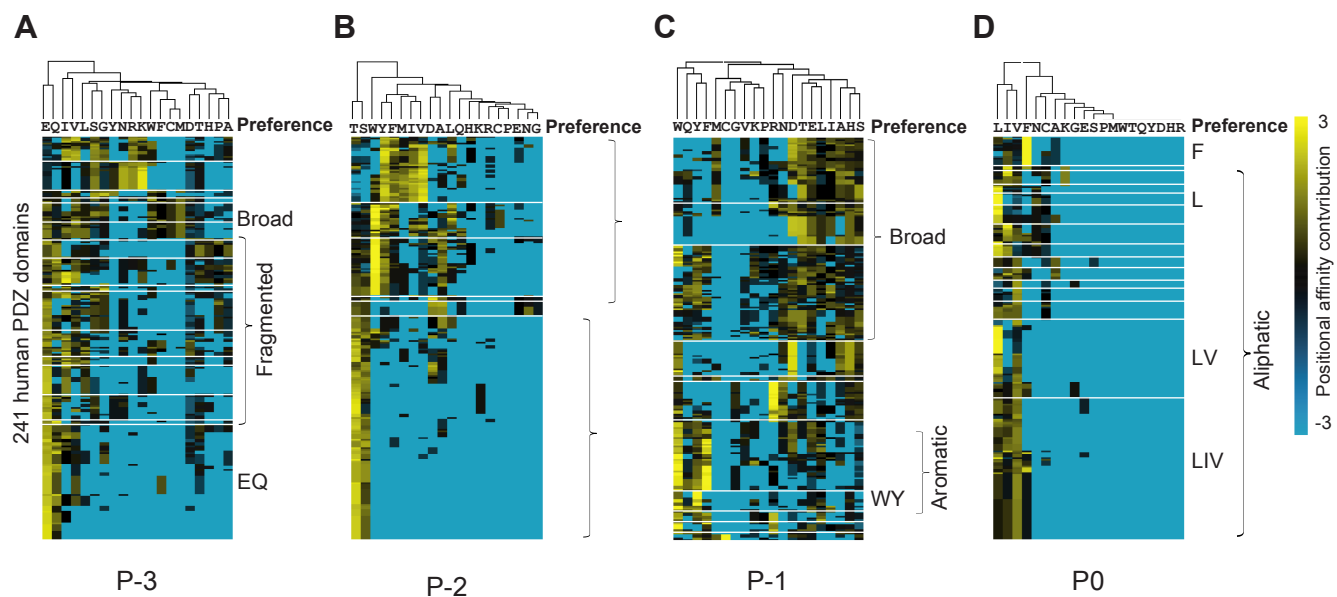

Supplement: Figure S8 — Amino acid preference patterns of human PDZ domain pockets. (A–D) Clustering of amino acid preference profiles of 241 human PDZ domain pockets is shown. (PDF) [file pgen.1002510.s008.pdf]

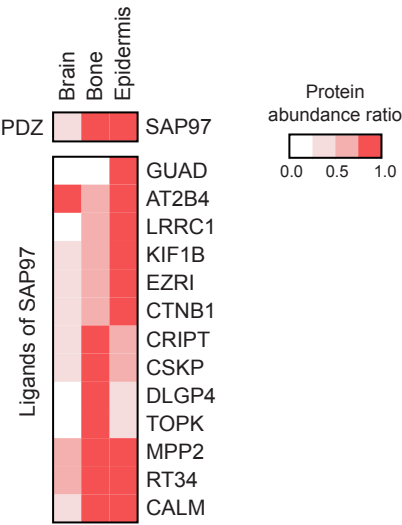

Supplement: Figure S9 — Alternative expression of SAP97 ligands across three human tissues. The protein expression levels of SAP97 PDZ protein and its 13 ligands were compared across brain, bone, and epidermis. Protein expression was measured by quantitative mass spectrometry [69]. The protein abundance ratio was defined by the normalized mass spectrometry intensity value relative to the maximum intensity per protein. (PDF) [file pgen.1002510.s009.pdf]

A

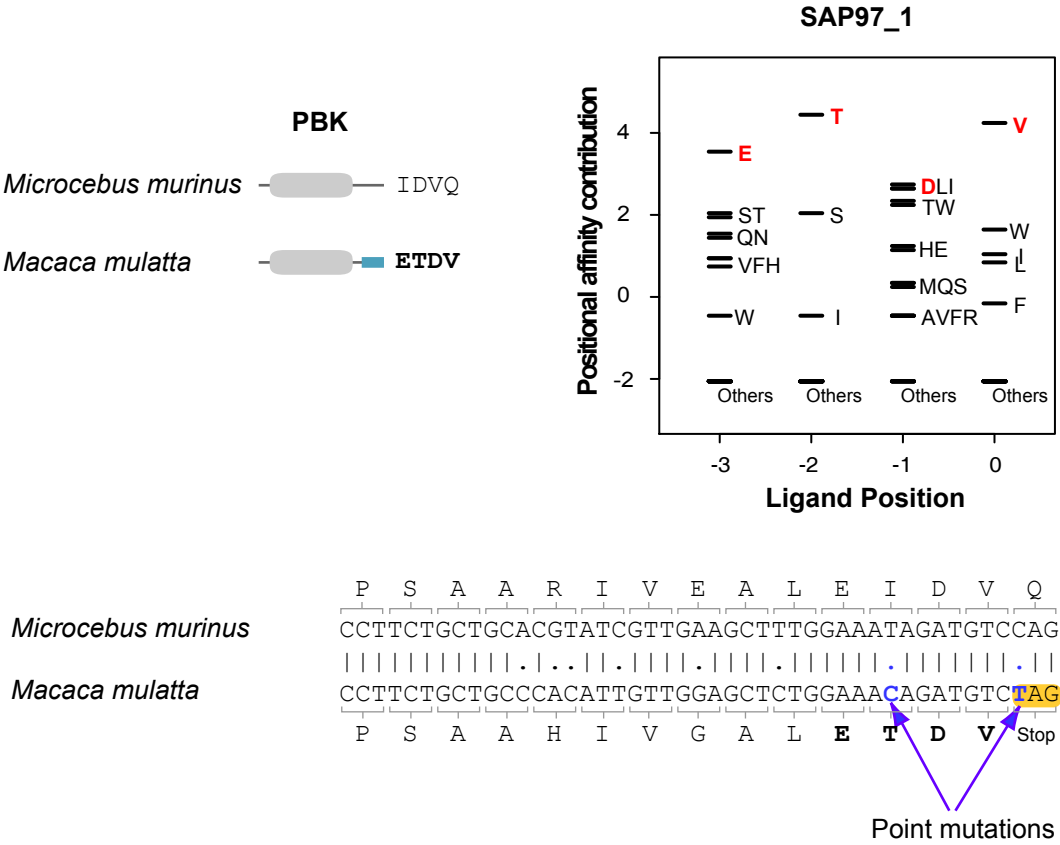

B

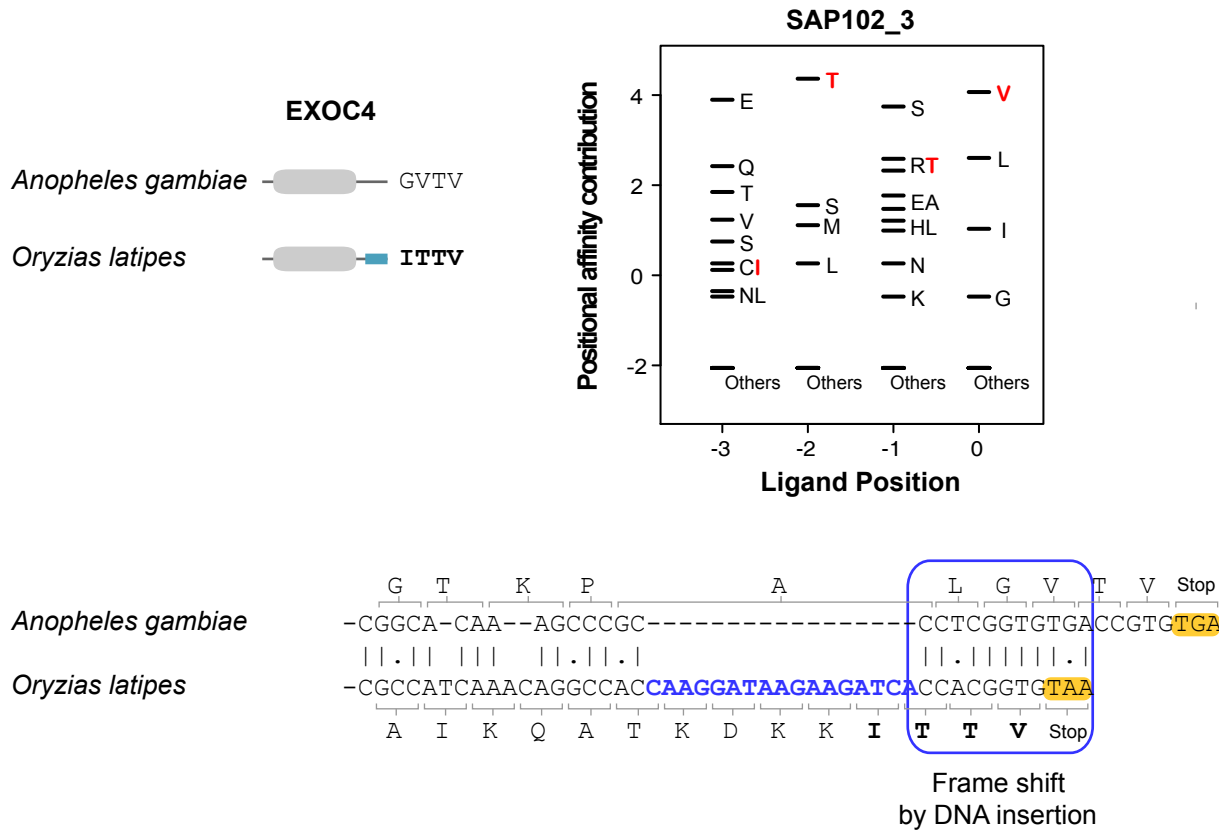

Supplement: Figure S10 — Types of DNA modifications that gain PDZ-binding motifs. (A) A point mutation generated a PDZ-binding motif in the C-terminal amino acids of the Macaca mulatta PBK protein. The binding motif is highlighted in the PWM of SAP97_1 (right). Mutations in the PDZ-binding motif are shown in the alignment of DNA sequences (bottom). (B) A DNA segment insertion generated a PDZ-binding motif in the C-terminal amino acids of the Oryzias latipes EXOC4 protein. The binding motif is highlighted in the PWM of SAP102_1 (right). The inserted DNA segment is shown in the alignment of DNA sequences (bottom). (PDF) [file pgen.1002510.s010.pdf]

A

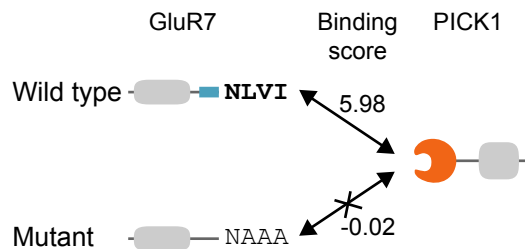

B

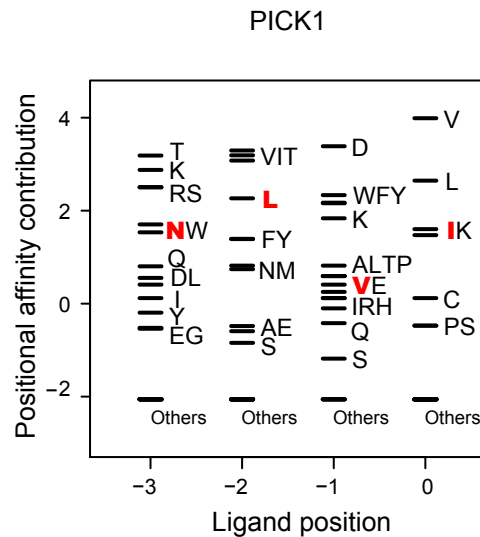

Supplement: Figure S11 — Mutation effects of the C-terminal GluR7 sequence. (A) C-terminal sequences and binding scores of wild-type and mutation forms of GluR7. (B) The PWM of the PICK1 PDZ domain. Four C-terminal residues of wild-type GluR7 are highlighted. (PDF) [file pgen.1002510.s011.pdf]

**A**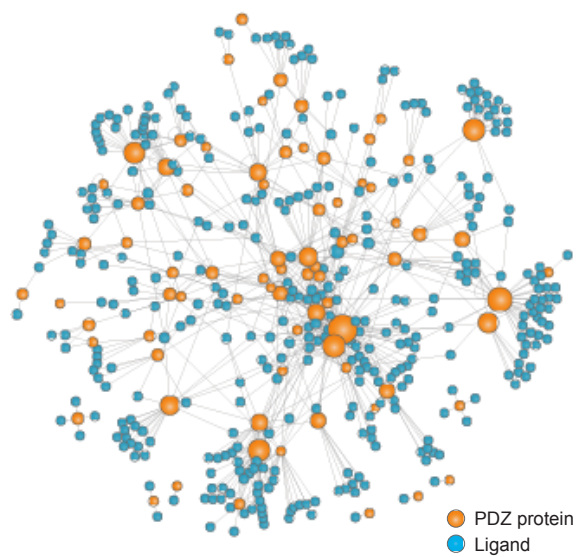**B**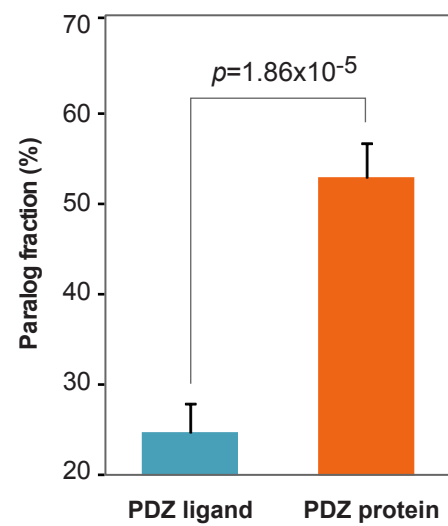

Supplement: Figure S12 — Repeated analysis of PDZNet by randomly removing 20% of proteins (trial 1). (A) Network representation of PDZNet. (B) Paralog fractions of PDZ ligands that share the same PDZ proteins (left) and PDZ proteins that share the same PDZ ligands (right). (PDF) [file pgen.1002510.s012.pdf]

**A**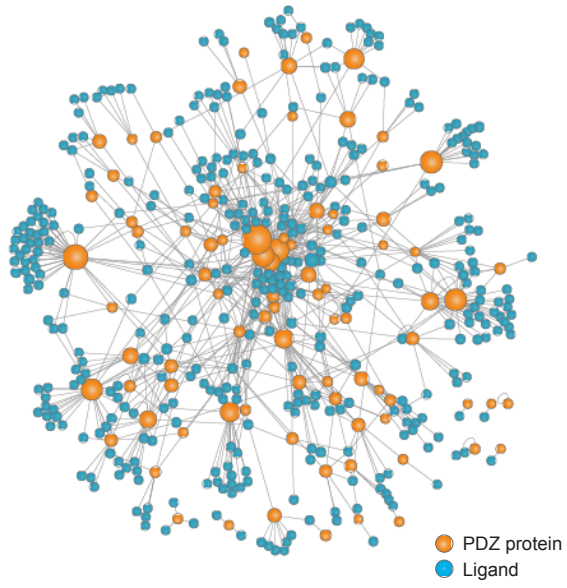**B**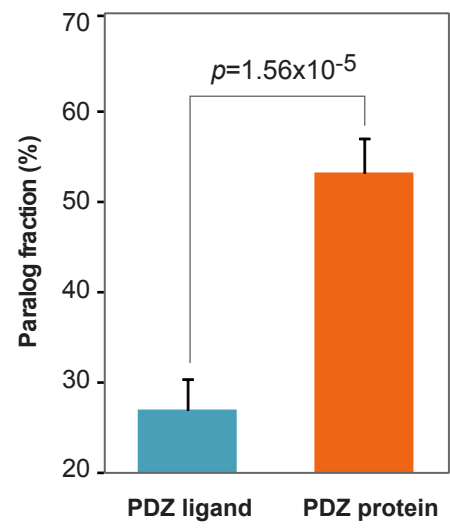

Supplement: Figure S13 — Repeated analysis of PDZNet by randomly removing 20% of proteins (trial 2). (A) Network representation of PDZNet. (B) Paralog fractions of PDZ ligands that share the same PDZ proteins (left) and PDZ proteins that share the same PDZ ligands (right). (PDF) [file pgen.1002510.s013.pdf]

**A**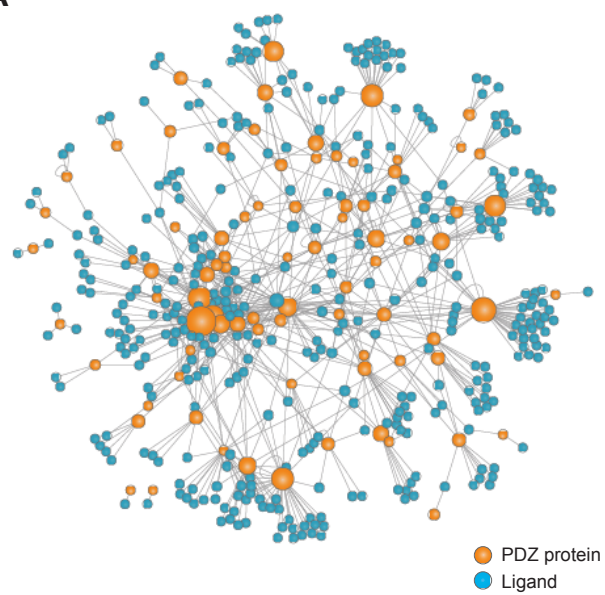**B**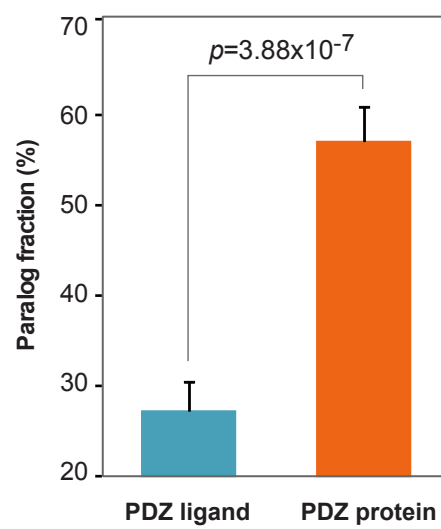

Supplement: Figure S14 — Repeated analysis of PDZNet by randomly removing 20% of proteins (trial 3). (A) Network representation of PDZNet. (B) Paralog fractions of PDZ ligands that share the same PDZ proteins (left) and PDZ proteins that share the same PDZ ligands (right). (PDF) [file pgen.1002510.s014.pdf]

**A**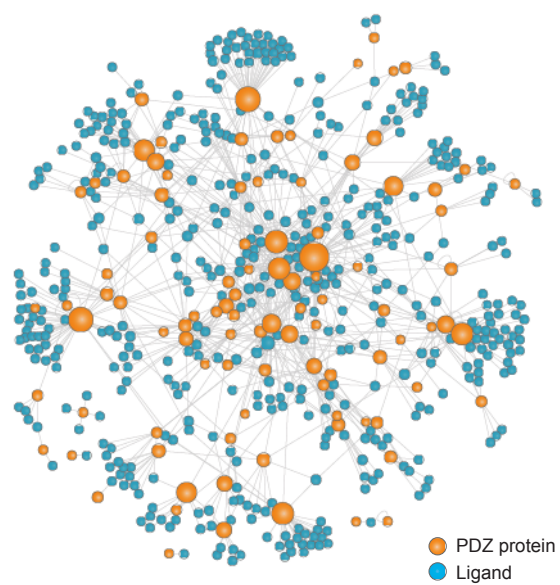**B**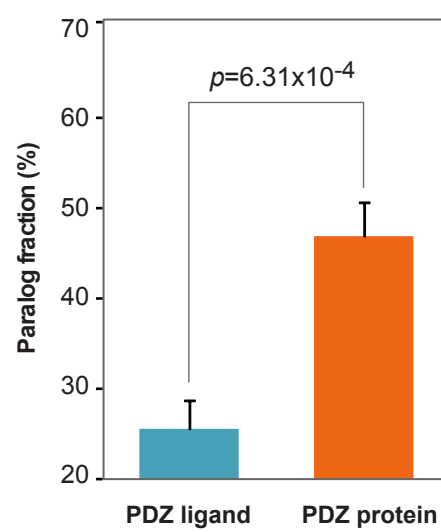

Supplement: Figure S15 — Repeated analysis of PDZNet by randomly removing 20% of interactions (trial 4). (A) Network representation of PDZNet. (B) Paralog fractions of PDZ ligands that share the same PDZ proteins (left) and PDZ proteins that share the same PDZ ligands (right). (PDF) [file pgen.1002510.s015.pdf]

**A**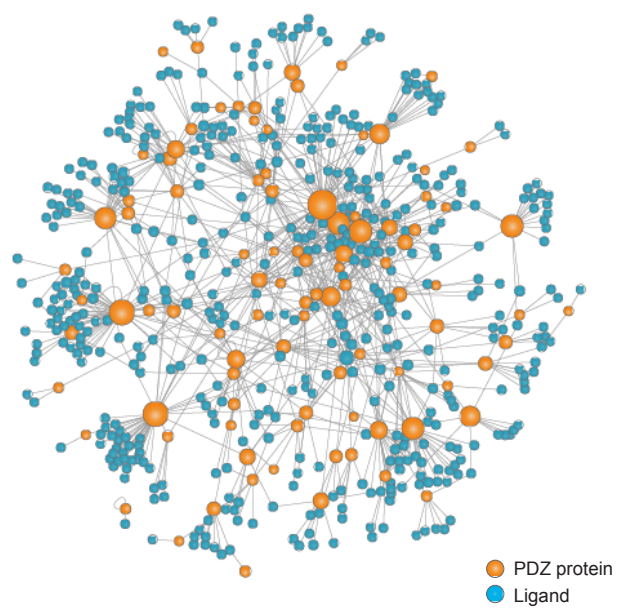**B**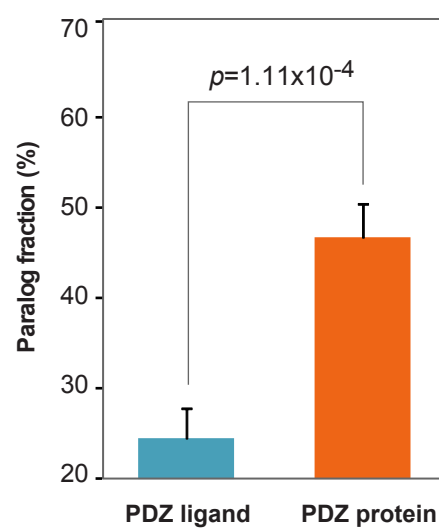

Supplement: Figure S16 — Repeated analysis of PDZNet by randomly removing 20% of interactions (trial 5). (A) Network representation of PDZNet. (B) Paralog fractions of PDZ ligands that share the same PDZ proteins (left) and PDZ proteins that share the same PDZ ligands (right). (PDF) [file pgen.1002510.s016.pdf]

**A**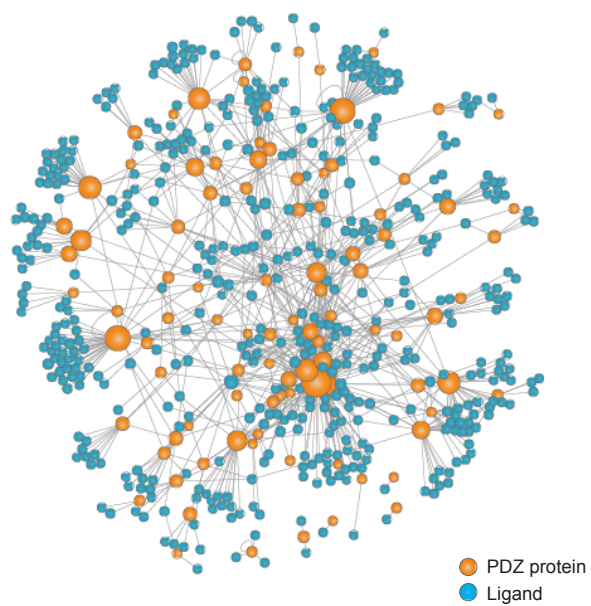**B**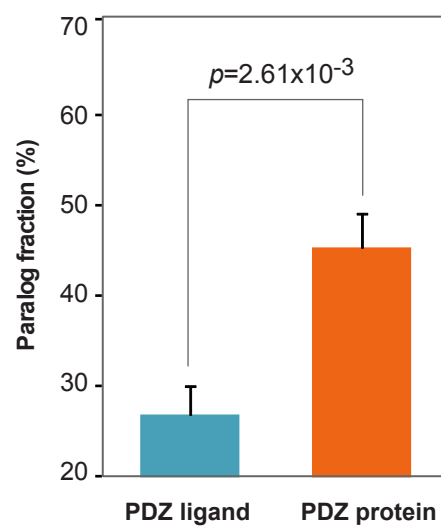

Supplement: Figure S17 — Repeated analysis of PDZNet by randomly removing 20% of interactions (trial 6). (A) Network representation of PDZNet. (B) Paralog fractions of PDZ ligands that share the same PDZ proteins (left) and PDZ proteins that share the same PDZ ligands (right). (PDF) [file pgen.1002510.s017.pdf]

A

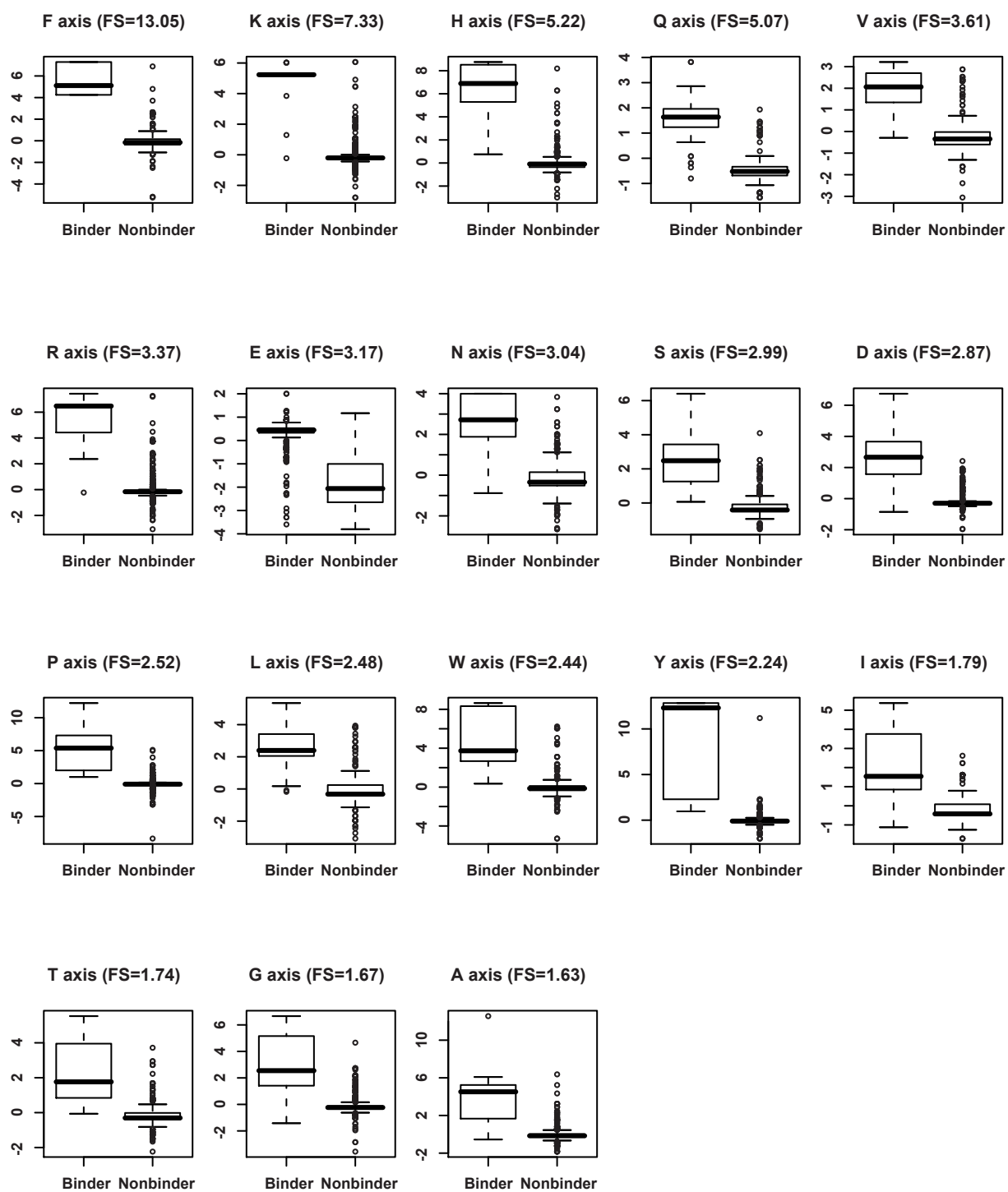

**B**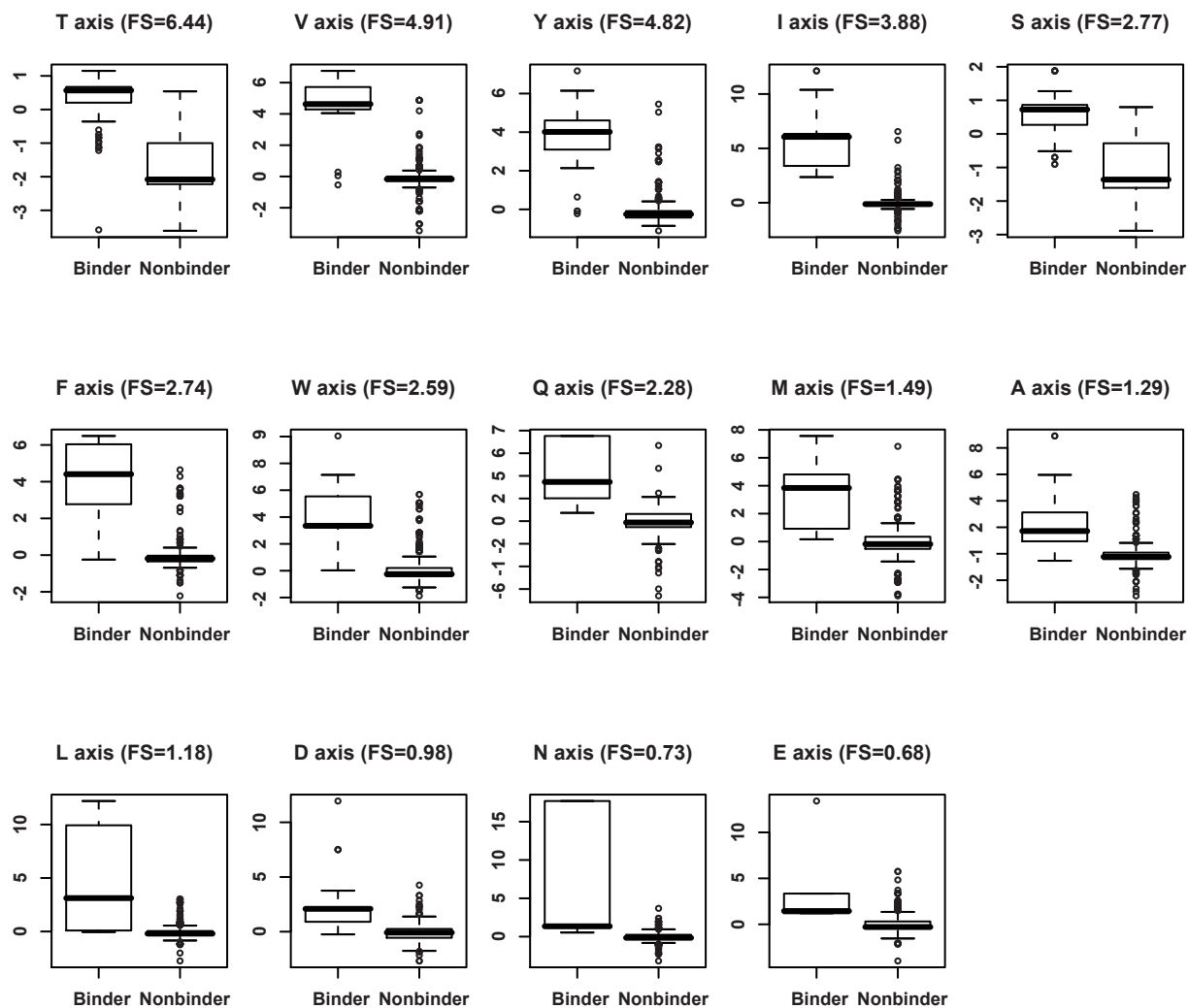

C

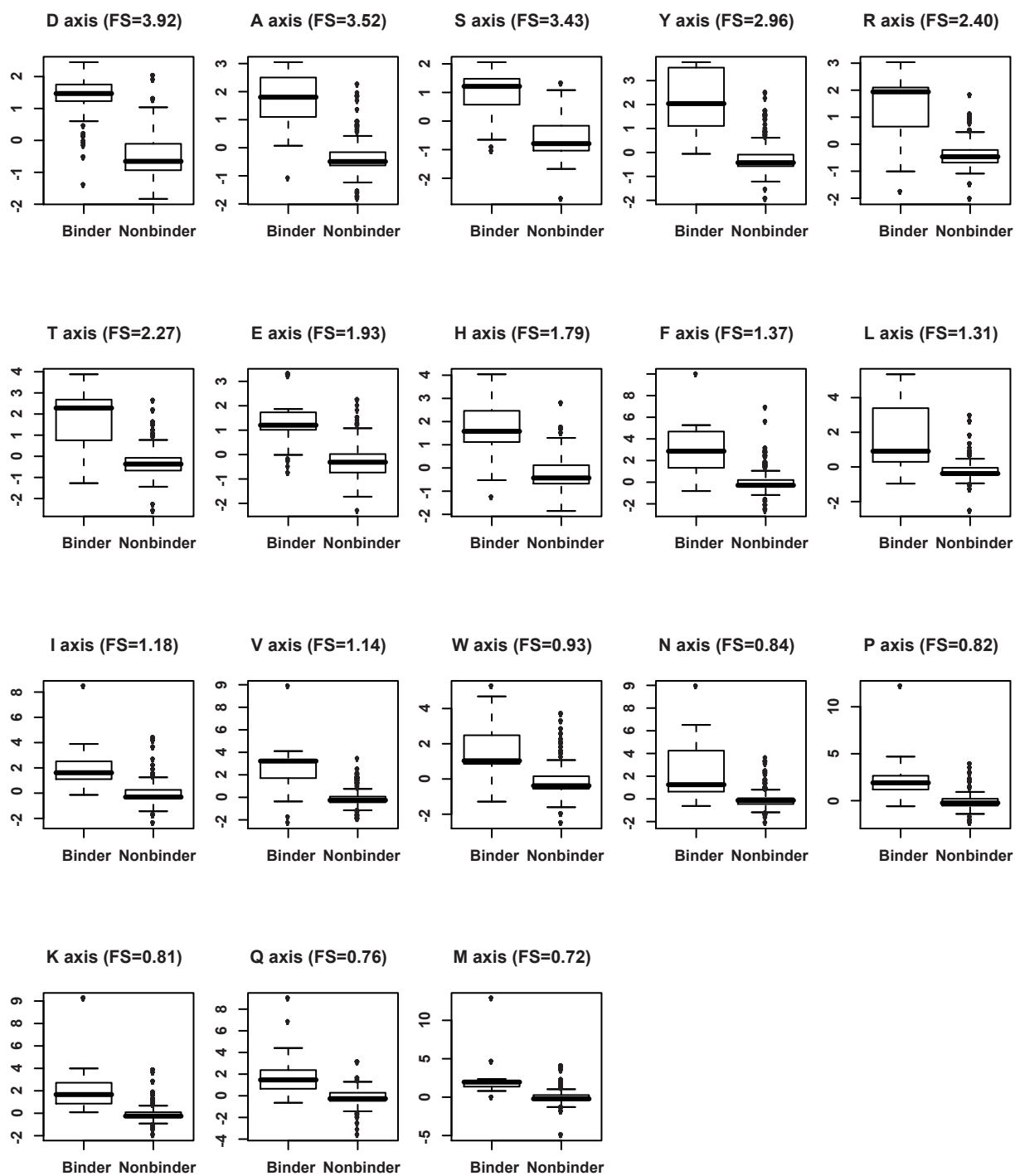

D

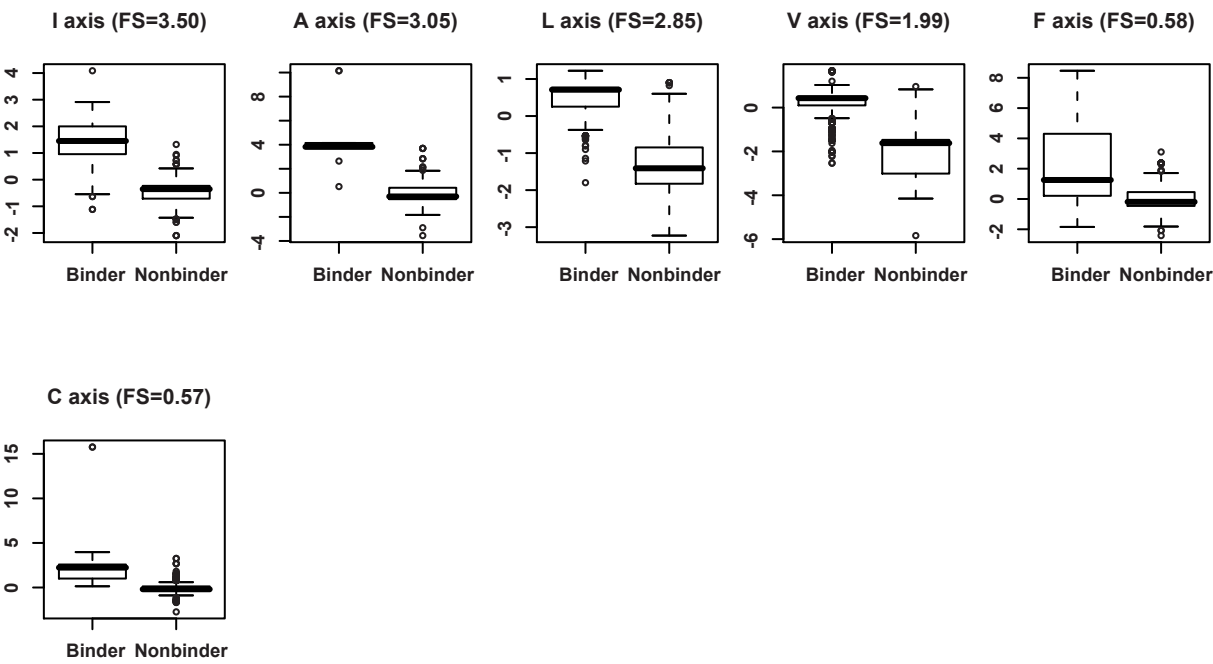

Supplement: Figure S18 — Discriminating power of selectivity axes. Each boxplot shows distributions of binders and non-binders of an amino acid, which are presented at the top of the plot. Binders are PDZ domain pockets that prefer the amino acid, and non-binders are those domain pockets that do not prefer the amino acid. The vertical axis corresponds to an axis of a selectivity space. Fisher's score (FS) is presented at the top of each plot, indicating the discriminating power of the selectivity axes. (PDF) [file pgen.1002510.s018.pdf]

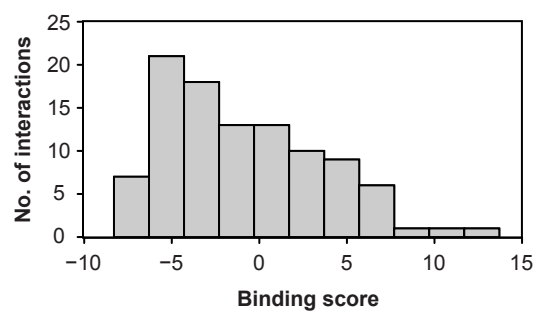

Supplement: Figure S20 — Fraction of domain-domain interactions according to the binding scores of all PDZ protein-mediated interactions. The PDZ protein-mediated interactions were binned based on binding score. The fraction of domain-domain interactions were measured for each bin. (PDF) [file pgen.1002510.s020.pdf]

Web server diagram and sample output

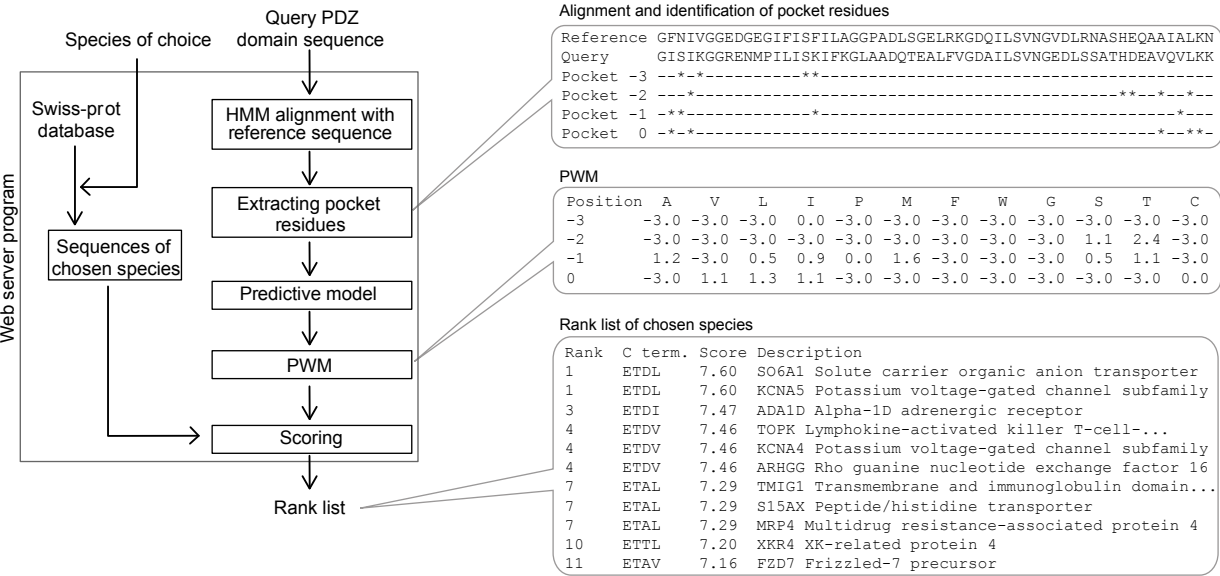

Supplement: Figure S21 — Flow chart of web server and a sample output. The web server takes a query PDZ domain sequence and a species name. The outputs are pocket residues, a PWM of the query PDZ domain, and a genome-wide rank list of proteins from the species chosen by the user. (PDF) [file pgen.1002510.s021.pdf]
