# Supplementary material for: Rewiring of PDZ Domain-Ligand Interaction Network Contributed to Eukaryotic Evolution
Source: PLoS Genet. 2012 Feb 9;8(2):e1002510. doi: 10.1371/journal.pgen.1002510 (PMC3276551; doi:10.1371/journal.pgen.1002510)
Supplement: Figure S7 — Multiple sequence alignment (MSA) of EXOC4 orthologs. The MSA was generated using Muscle with default options. C-terminal PDZ binding motifs are shown in bold. (PDF) [file pgen.1002510.s007.pdf]

```
Yeast     -----MDYLKPAQKGRRRGLSINSLSETQQSAMNSSLDHLQNDLNRINLQWNRILSDNTNPLELALAFLDDTSVGLGHRYEEFNQLKSQIGSHLQDVVNEHSQVFNTNVASYGKAVSSIM
Worm      MNENGATPVAAARRHRPLPAERATSNSNETGLLINVIRSLTSSVS----------------------EDQREVEKSRLEKGYKE----SGALIDRLVKNHQQDVEKCLVSFRDVSSKIS
Mosquito  --------MDSPPVKPPRGVKY-TKETSGCGLLVSVIKTLDASET----------------------NEQREREKLKIEREFRK----TDQRLNELVSRSDGDLTRVMQLFGKVSTEVT
Fly       -------MDAPPPTKPPRGVKYGKDESAGCGFLVNVIKSLGFSET----------------------TEERQKEKQKIEAEFKR----SDLRLNELVSRHDQQLTQVLPLFSQVSSEVT
Chicken   -------MAAGEAVGRYRSSM--SRSKDPSGLLISVIRTLSTSDD----------------------VEDRENEKGRLEEAYEK----CDRDLDELIVQHYTELTTAIRTYQSITERIT
Mouse     -------MAAEAAGGKYRSTV--SKSKDPSGLLISVIRTLSTSDD----------------------VEDRENEKGRLEEAYEK----CDRDLDELIVQHYTELTTAIRTYQSITERIT
Rat       -------MAAEAAGGKYRSTV--SKSKDPSGLLISVIRTLSTSDD----------------------VEDRENEKGRLEEAYEK----CDRDLDELIVQHYTELTTAIRTYQSITERIT
Human     -------MAAEAAGGKYRSTV--SKSKDPSGLLISVIRTLSTSDD----------------------VEDRENEKGRLEEAYEK----CDRDLDELIVQHYTELTTAIRTYQSITERIT

Yeast     QAQEQTLNLKNCLKEANEKITTDKGSLQELNDNNLKYTKMIDVLVNIEELLQIPEKIEENIRKENFHQVQILLERGFILMNNKSLKTVEILKPINQQLELQEHLLFNNLIEEIHDIMYSK
Worm      NCRERIHNVRNALHTVKSLLELRRDDLKKLWHENAQQKSVCEIMAKLEELREAPSKIENLISKEQYQQAADTVTESRELINGR-LSRVEGLSHLSAEIERFTKILIDKINDTLVNMLVVE
Mosquito  ASRERIQVVKENLQSCKQLLRCRRDELKKLYTDAIQHKYVLEMLDQITEIRKVPAQLTAFMAKKHYLHATKLLMASIETTDGK-LKGVEGLNDLRQDFQNRRQQLYGKLLEELNKHLYVS
Fly       ASRERIHAVKENLGVCKRLLQCRRDELRKMWMDAVQHKYVLEMLEQIQELRKVPQRVVGYTAKRQYLHASKALTDALTTLNGP-LQAVEGLSDLRTDLQTRRQQLYQRLHEELVTQVYTN
Chicken   NSRNKIKQVKENLLSCKMLLHCKRDELRKLWIEGIEHKHVLNLLDEIENIKQVPQKLEQCMASKHYLNATDMLVSAVDSLEGP-LLQVEGLSDLRLELHSKKMNMHLVLIDELHRHLYIK
Mouse     NSRNKIKQVKENLLSCKMLLHCKRDELRKLWIEGIEHKHVLNLLDEIENIKQVPQKLEQCMASKHYLSATDMLVSAVESLEGP-LLQVEGLSDLRLELHSKKMNLHLVLIEELHRHLYIK
Rat       NSRNKIKQVKENLLSCKMLLHCKRDELRKLWIEGIEHKHVLNLLDEIENIKQVPQKLEQCMASKHYLSATDMLVSAVESLEGP-LLQVEGLSDLRLELHSKKMNLHLVLIEELHRHLYIK
Human     NSRNKIKQVKENLLSCKMLLHCKRDELRKLWIEGIEHKHVLNLLDEIENIKQVPQKLEQCMASKHYLSATDMLVSAVESLEGP-LLQVEGLSDLRLELHSKKMNLHLVLIDELHRHLYIK

Yeast     SNKTNFTRVTNNDIFKIISISHNGFTSLENYLYNIVNIDIMEHSKTINKNLEQFIHDQSLNKGNIMLQENAATQAPLAPSRNQENEGFNRIGFLLKTINNINKLPVAFNIITERAKEEIH
Worm      PFEKHLL-----------HIVRTIPEHRI---NQ-NAYCHSLSTKSRSGSGSVS-------SFSDVSAKSRI----------VSSVEALSTLFRTVEERHWDVDRLMMLGKNMIDKMI
Mosquito  STADVLQNFQRQASGRNSQYAAGTSPFQRNVLRR--SAERVEANTKARKALFEISKNGFLDVDKSEIIEDTELLDP-------DVNSSYFLSIIVECFALLQKVPESIESIKVQMQSELT
Fly       SANEALSSFQRTNSSRLNSSFTRGIGARR-------STDRIEANARVRKALAEMAQS--FDLDKAEVIEDADLIYP-------ELSMSYFVAIIVESFGMLHKVPDSLETLRVQIQTEL-
Chicken   STNKVGQ--RNKEKGRISSLVKDASSVPLMDVTNL-STPRKFLETSQFSTPGSSSMR---ETSLLEIKEDMEL-DP-------EENSTIFMGILIKGLAKLKKIPETVKAIQDRLEQEL-
Mouse     STSRVVQ--RNKEKGKMSSHGKDPSPGPLIDVSNI-PTPRKFLDASQYSAAGGSSVR---EMNLQDVKEDLEC-DP-------EENSTLFMGILIQGLARLKKIPETVKAIKERLEQEL-
Rat       STSRVVQ--RNKEKGKMSSHGKDASPGPLIDVSNI-STPRKFLDATQYSAAGSSSVR---EMNLQDIKEDLDC-DP-------EENSTLFMGILIQGLARLKKIPETVKAIKERLEQEL-
Human     STSRVVQ--RNKEKGKISSLVKDA-SVPLIDVTNL-PTPRKFLDTSHYSTAGSSSVR---EINLQDIKEDLEL-DP-------EENSTLFMGILIKGLAKLKKIPETVKAIIERLEQEL-

Yeast     NIIVKSTESIRSKHPSLLKMATSLKNDNHFGLPVQDILSIILRECFWEIFLKLLYAIQCHRAIFEMSNILQPTSSAKPA-----FKFNKIWGKLLDEIELLLVRYINDPELISSNN--GS
Worm      VNTVQVMKIGANIDE-------SNEGDTT---HLKQLMQL--------LSAQFDSASQQHA----EFGVLVEKQLGRKD------VVTSFWRSAQSAIEVVVSEHLDI------------
Mosquito  VATCNGTPSGASSAD------PTQPAQPV---PILELLDL--------VFKQFKLIANAHQQALRNYGNVI-QRYNIPQ--VKPYDIIEYWGQAQAVLKLVLTDYLDIQNDSGDEALLLR
Fly       LNVVRHTTHQLSVSG--------ATADTN---PLLSLLEV--------IFKQFKAIAKTHSLLLKNY-LSVGQKYSVVG--PQPYDLTDFWAQAQSVLQLLLTDYLDIQNAAADES--AQ
Chicken   KQIVKRSTTQVADSDYQRGENISQENQPR---LLLELLEL--------LFDKFNAVAAAHSVVLGHLQQTVASPCSQYDGDIKLYDMVDVWVKIQDVLQMLLTEYLDMKNTRTASEPSTQ
Mouse     KQIVKRSTTQVADSAYQRGESLTVDNQPR---LLLELLEL--------LFDKFNAVATAHSVVLGYLQDSVGTQLTQQE-EIKLYDMADVWVKIQDVLQMLLTEYLDMKNTRTASEPSAQ
Rat       KQIVRVSMTSTSESALKQGQASSLDHQQR---LLLELLEL--------LFDKFNAVASAHSIVLGYLQDSVGTQPTQQE-EIKLYDMADVWVKIQDVLQMLLTEYLDMKNTRTASEPSAQ
Human     KQIVKRSTTQVADSGYQRGENVTVENQPR---LLLELLEL--------LFDKFNAVAAAHSVVLGYLQDTVVTPLTQQE-DIKLYDMADVWVKIQDVLQMLLTEYLDMKNTRTASEPSAQ

Yeast     IKPINGATNNAPTLPKRKNPKI--FSLEYNIEDNSS---------VKDQAFELKALLKDIF----------------PGFSVSSNMDLDSIYVKDESFEQDEPLVPPSVFNMKVILDPF
Worm      ----------NPLLEKQNVLGTASRKQLFRFENTAC--------------------------------------------ATPNANS-------SSHRTKAVICKPSAYNIKVIFPIL
Mosquito  GQFAEQSTNVNAFFSRRKTPG---KKLLFRFDKSSHTADAGGPQTADGDTGGTKEHRRNLSNVSNSTPKEEALLNSSAPYYFGGKDAAA------ERRKRERALVCPADASLVRKIYLPL
Fly       TGFSEPTSNINSYFLRRKVPST--KRSMFKFDKSSHVG-------TSNNSDAFKEHRRNASDASVD--------DNLAGQLGGSGKGSTSGLFPHEKKQREKILICTPDQSIITKVYLPL
Chicken   LSYASSGREFAAFFAKKKPQRP--KNPLFKFESSSH---------AISMSAYLREQRRELY--------------SRSGELQGGPDDNL-------IEGGGSKFVCKPGARNITIIFHPL
Mouse     LSYASTGREFAAFFAKKKPQRP--KNSLFKFESSSH---------AISMSAYLREQRRELY--------------SRSGELQGGPDDNL-------IEGGGTKFVCKPGARNITVIFHPL
Rat       LSYASTGREFAAFFAKKKPQRP--KNSLFKFESSSH---------AISMSAYLREQRRELY--------------SRSGELQGGPDDNL-------IEGGGTKFVCKPGARNITVIFHPL
Human     LSYASTGREFAAFFAKKKPQRP--KNSLFKFESSSH---------AISMSAYLREQRRELY--------------SRSGELQGGPDDNL-------IEGGGTKFVCKPGARNITVIFHPL

Yeast     LLFTQSTSTIVPSVLTQNTISSLTFFDDYMNKSFLPKIQM-TMDYLFTVEVESNNPYALELSDE-------NHNIFKTALDFQRLFYNLLNVFNTANTFREKISYCILDLLNHFYNYYLG
Worm      SRLMETTEKNINDSP----CELRRFMHSFVMEVFVERVKG-ELASRIEGALRGGEAVRVSTN----------KKILPSCEKVLNLCKEVQDLIVSIDLYADRFAALWLLVLTDYFKNMTD
Mosquito  MGYIQEIESFMKCKSGQP-CSLNTFLANYVKDAYLARGHNRNLQLTIESLSKAQDAWRTIISPEEMKRLGLTRPLLQNTLIVENRIAETKKLIQDLPTYSDELLKMVCSLLKTYRETCQA
Fly       MGYIKEIENFMKCKPGQP-CSLHDFLDNYIKDTFLTKGHNRNLQLTIESLSKNQDAWRTIISPEEIKALNLSRPLLQSTVMVERRLMETKNLIQDLPCYSDELLKMVCALLKAYREICQA
Chicken   LRFIKEIEHAMELGPTKQ-CPLREFLTMYIKNIFLNQVLA-EINKEIEGVTKASDPLKILANADTMKVLGVQRPLLQSTVIVEKTVQDLMNLMHDLSAYSDQFLNMVCVKLQEYKDTCTL
Mouse     LRFIQEIEHALGLGPAKQ-CPLREFLTVYIKSIFLNQVLA-EINKEIEGVTKTSDPLKILANADTMKVLGVQRPLLQSTIIVEKTVQDLMNLMHDLSAYSDQFLNMVCVKLQEYKDTCST
Rat       LRFIQEIEHALGLGPAKQ-CPLREFLTIYIKNIFLNQVLT-EINKEIEGVTKTSDPLKILANADTMKVLGVQRPLLQSTIIVEKTVQDLMNLMHDLSAYSDQFLNMVCVKLQEYKDTCST
Human     LRFIQEIEHALGLGPAKQ-CPLREFLTVYIKNIFLNQVLA-EINKEIEGVTKTSDPLKILANADTMKVLGVQRPLLQSTIIVEKTVQDLLNLMHDLSAYSDQFLNMVCVKLQEYKDTCTA

Yeast     LFNSLIGTSDRH---------LTRKIITA-WLQNGILMDQEQKILNGDETLFHEESIELFK------------EIPHFYQAGKGLSKSDLFNNLT---LDTILQFSASVLWILNWLPGLK
Worm      VYDRMTPKNPDPSLPNSEALPTRRQKISAAWTADDDISRLLMSLPNWHAASISPMTPAV--------------ESELDVGERNKRESEILIGNLGTQAQNSLSESD-----LITDMNDIK
Mosquito  AYRGIVQPETED-----------KRIYSVAWLKDDDITRFLKSLPNWTDLKSSNARLRQLQQKRLMKTEPSEEESPTQVQQRNVREAEMLTSNLG---EGGISQQE-----ILSDVGVLK
Fly       AYRGIVQPDSED-----------KRIYSVAWLKDEDISRFLKTLPNWTDLKTYSQKSRHNRKLHRGSFEPSEEESPLQVQQRNIREAEMLTSNLG---EGGITQQE-----ILVEISVLK
Chicken   AYRSIVQSDE-------------KLVISASWAKDDDISRLLKSLPNWSNM----AQPKQLR--------PKREEEEDFIRAAFGKESEVLIGNLG---DKLIPQQE-----ILRDVSDLK
Mouse     AYRGIVQSEE-------------KLVISASWAKDDDISRLLKSLPNWTNM----AQPKQLR--------PKREEEEDFIRAAFGKESEVLIGNLG---DKLIPPQD-----ILRDVSDLK
Rat       AYRGIVQSEE-------------KLVISASWAKDDDISRLLKSLPNWTNM----AQPKQLR--------PKREEEEDFIRAAFGKESEVLIGNLG---DKLIPPQD-----ILRDVSDLK
Human     AYRGIVQSEE-------------KLVISASWAKDDDISRLLKSLPNWMNM----AQPKQLR--------PKREEEEDFIRAAFGKESEVLIGNLG---DKLIPPQD-----ILRDVSDLK

Yeast     KAINIDEVSQEPMLDADRLRSSWTFSESMDLNY--------SNPSSSPNSLGLNKLKILLDDKASKKFDETIDGFKTLKFKLITILRFNIRALCIYDIGSF--FQNTKIWNMDVGSIELDQN
Worm      MFASLHESLRWFSDEIRELVHSLPANVKMML-----------DTCMVQVRLKDGQMIDNNSVPSAIEDCVRRLESIADSCLLLLHIEIRVHCFFHLAPLAKYRNTSSHN------EVDPE
Mosquito  ELAILQESMEWFASRITEFAHDLKKPIVNGLVAASPVTTPGSPSPAGTATIVNSPVIVKDGMIKVLINLALEFEELANTCLLVLHLEVRVQCFHYLRSSPSDRYKANNPNKNDSLEPDAK
Fly       ELAILQESMEWFSCRVSEFANDLRRPLVNGLNA--------VPAECG------ADIAVKDGTIKVMTNLALEFDELANTCLLVLHLEVRVQCFHYLRSKSSVRTNSYVGSKDDILEPDRQ
Chicken   ALANMHESLEWLAGRTKAAFSNLSASQTMS------------PGQDSHASMENLP--ASEQILQTLSELARSFQEMADRCLLVLHLEVRVHCFHYLIPLAKQGNYAIVA-NVESMDYDPL
Mouse     ALANMHESLEWLAGRTKSAFSNLSTSQMLS------------PAQESHVNMDLPP--VSEQIMQTLSELAKTFQDMADRCLLVLHLEVRVHCFHYLIPLAKEGNYAIVA-NVESMDYDPL
Rat       ALANMHESLEWLAGRTKSAFSSLSASQMLS------------PAQESHVNMDLPP--VSEQIMQTLSELAKSFQDMADRCLLVLHLEVRVHCFHYLIPLAKEGNYAIVA-NVESMDYDPL
Human     ALANMHESLEWLASRTKSAFSNLSTSQMLS------------PAQDSHTNTDLPP--VSEQIMQTLSELAKSFQDMADRCLLVLHLEVRVHCFHYLIPLAKEGNYAIVA-NVESMDYDPL

Yeast     IASLISELRRTESKLKQQLPEKEKNSIFIGLDIVNNYALIKGAKSIKVLNHNGIKKMLRNVNVLQHAYRNLSSEPSKINMNVTMNFYSLCGSSEAELFEYIKDNELPHCSVEDLKTILRL
Worm      VVALGKDLHQFHDNLKDVLSPAKLSYVFDGLGHLCASLFIHYSQFMPRLTEAAKKRVCRNVWGVQQRLSRITN-RRESDLDRARAFF--------DLLLDNTPDGILAIVPEKRSQFTAT
Mosquito  VLKLTKVLSDMDEALSSTLHPRKTKYVFEGLAHLAARILIMAANSMEYIDHSGVQRMCRNALALQQTLSSITA-SREVALDYARSFY--------EMF-YLDPEEILTSIVEKGAQFTEM
Fly       VQVLTKRLSEMDEAFSATLHPRKTRYIFEGLAHLASRILIQASNYLEHIDQITVQRMCRNAIALQQTLSNITA-SREVALDQARHFY--------ELL-CMEPDEILNALLERGTQFSEM
Chicken   VVRLNKDISAIEEAMSASLQQHKFQYIFEGLGHLISCILINGAHYFKRISESGIKKMCRNIFILQQNLTNITM-SREADLDFARQYY--------EML-YNTVDELLNLEVDQDLKYTGG
Mouse     VVKLNKDISAMEEAMSASLQQHKFQYIFEGLGHLISCILINGAQYFRRISESGIKKMCRNIFVLQQNLTNITM-SREADLDFARQYY--------EML-YNTADELLNLVVDQGVKYTEL
Rat       VVKLNKDISAMEEAMSASLQQHKFQYIFEGLGHLISCILINGAQYFRRISESGIKKMCRNIFVLQQNLTNITM-SREADLDFARQYY--------EML-YNTADELLNLVVDQGVKYTEL
Human     VVKLNKDISAIEEAMSASLQQHKFQYIFEGLGHLISCILINGAQYFRRISESGIKKMCRNIFVLQQNLTNITM-SREADLDFARQYY--------EML-YNTADELLNLVVDQGVKYTEL

Yeast     QFSEEMHRQLKRQSTS-STKGSIKPSNKRYTEALEKLSNLEKEQSKEGARTKIGKLKSKLNAVHTANEK
Worm      ELNYLLALSVRSDKTLASQPGALEKRQMVLNSIL-------------NQKK------------------
Mosquito  QYLNALQLIFRNR-GI-VDPAVVGTYQQKLSDLL-------------GTKPALGVTV------------
Fly       QLLNALQLSCKSF-GI-TDANLLASYQQKLSDIL-------------GAKPSKGVVV------------
Chicken   EYIYAMDLLRLSQTGV-GDQTTRNMRLQRRKEII-----CEQAAIKQATKDKKITTV------------
Mouse     EYIHALTLLHRSQTGV-GDQTTQNTRLQRLKEII-----CEQAAIKQATKDKKITTV------------
Rat       EYIHALTLLHRSQTGV-GDQTTQNTRLQRLKEII-----CEQAAIKQATKDKKITTV------------
Human     EYIHALTLLHRSQTGV-GELTTQNTRLQRLKEII-----CEQAAIKQATKDKKITTV------------
```
