# Supplementary material for: Rewiring of PDZ Domain-Ligand Interaction Network Contributed to Eukaryotic Evolution
Source: PLoS Genet. 2012 Feb 9;8(2):e1002510. doi: 10.1371/journal.pgen.1002510 (PMC3276551; doi:10.1371/journal.pgen.1002510)

A

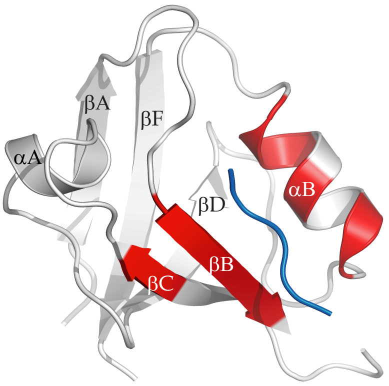

B

Ligand Position	Pocket Residues
**P -3**	βB2 βB4 βC4 βC5
**P -2**	βB3 αB1 αB2 αB5 αB8
**P -1**	αB7 βB1 βB2 βC4 βC5
**P 0**	αB9 βB1 βB3 αB5 αB8

C

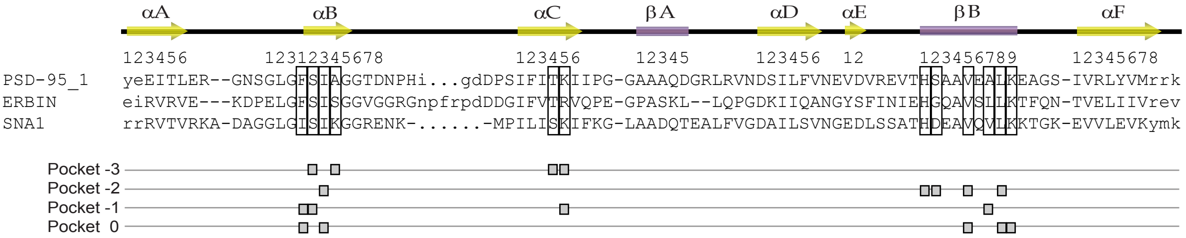

Supplement: Figure S19 — Procedure for extracting pocket residues. (A) Schematic drawing of the PSD-95_1 domain structure. (B) Position of each pocket residue on the structure. (C) The MSA of three representative PDZ domains was constructed using the hidden Markov model that was optimized for the PDZ domain. By adjusting the secondary structural profile on the MSA, the positions of pocket residues were identified. Gray boxes indicate the positions of pocket residues. (PDF) [file pgen.1002510.s019.pdf]
